# In situ structure of the *Caulobacter crescentus* flagellar motor and visualization of binding of a CheY‐homolog

**DOI:** 10.1111/mmi.14525

**Published:** 2020-05-25

**Authors:** Florian M. Rossmann, Isabelle Hug, Matteo Sangermani, Urs Jenal, Morgan Beeby

**Affiliations:** ^1^ Department of Life Sciences Imperial College London London UK; ^2^ Focal Area of Infection Biology Biozentrum of the University of Basel Basel Switzerland

**Keywords:** *Caulobacter crescentus*, CheY, cyclic‐di‐GMP, effector binding, subtomogram averaging

## Abstract

Bacterial flagellar motility is controlled by the binding of CheY proteins to the cytoplasmic switch complex of the flagellar motor, resulting in changes in swimming speed or direction. Despite its importance for motor function, structural information about the interaction between effector proteins and the motor are scarce. To address this gap in knowledge, we used electron cryotomography and subtomogram averaging to visualize such interactions inside *Caulobacter crescentus* cells. In *C. crescentus*, several CheY homologs regulate motor function for different aspects of the bacterial lifestyle. We used subtomogram averaging to image binding of the CheY family protein CleD to the cytoplasmic Cring switch complex, the control center of the flagellar motor. This unambiguously confirmed the orientation of the motor switch protein FliM and the binding of a member of the CheY protein family to the outside rim of the C ring. We also uncovered previously unknown structural elaborations of the alphaproteobacterial flagellar motor, including two novel periplasmic ring structures, and the stator ring harboring eleven stator units, adding to our growing catalog of bacterial flagellar diversity.

## Graphical abstract

The flagellar motor is a tiny bacterial nanomachine that needs to carefully respond to the bacterium's surroundings. We determined the first molecular structure of a flagellar motor with a bound regulatory protein inside a living *Caulobacter crescentus* cell. We also discovered intriguing new features of the flagellar motor from this bacterium with unknown roles. Our findings will help us to understand how nanomachines can be controlled at a molecular level.

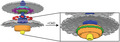

## 1 INTRODUCTION

Bacterial flagella are propulsive helical filaments rotated by intricate nanomachines for motility, which quickly respond to environmental conditions by binding various effector proteins. Effector binding facilitates effective swimming motility, swarming motility (Jarrell and McBride, 2008) and surface sensing in the early phases of biofilm formation (Hug *et al*., 2017; Laventie *et al*., 2019). The mechanistic core of these motors is a “gearbox” that drives and modulates flagellar rotation, composed of a rotating rotor and an immobilized stator (Blair *et al*., 2008; Pilizota *et al*., 2009; Stock *et al*., 2012). The stator is a ring of inner‐membrane stator complexes anchored to peptidoglycan; individual stator complexes are ion channels, which couple proton flux to drive rotor rotation. Torque is generated by electrostatic interactions with a cytoplasmic ring (“C ring”) lying beneath the stator as part of the rotor. The C ring is composed of three proteins, FliG, FliM, and FliN that act as a central hub for effector protein binding. The structural basis of effector binding, however, has never been imaged in situ; understanding effector binding would contribute to our understanding of the mechanism of signal response to the bacterial flagellar motor. In enteric bacteria, additional ring‐like structures, called the MS‐, P‐ and L rings span the inner membrane, the peptidoglycan layer and the outer membrane. The MS ring serves as assembly platform for the cytoplasmic part of the motor and transmits the torque from FliG to the rod. P‐ and L‐ rings act as bushings for the axial driveshaft which transmits the torque to the extracellular flagellum (Berg, 2003; Morimoto and Minamino, 2014; Minamino and Imada, 2015; Beeby *et al*., 2020).

The chemotaxis response regulator CheY is the best studied effector protein directly interacting with the C ring. Extracellular changes of attractant and repellent concentrations are sensed by chemoreceptors, which alter the phosphorylation state of CheY. This allows binding to a conserved motif present at the N‐terminus of FliM (Lee *et al*., 2001; Dyer *et al*., 2004; Dyer and Dahlquist, 2006), enabling “navigation,” or chemotaxis, toward attractants and away from repellents. CheY‐P‐binding is thought to induce large conformational changes in the C ring between FliG and the stator complexes, leading to reversal of flagellar rotation. In the model bacteria *Escherichia coli* and *Salmonella enterica* Serovar Typhimurium with flagella situated randomly over the cell body, rotation switches from a counter‐clockwise (CCW) to a clockwise (CW) direction to trigger a random cell reorientation by tumbling (Bren and Eisenbach, 1998; Stock *et al*., 2012). In polar flagellates like *Campylobacter, Vibrio, Shewanella, or Caulobacter* species, motor reversal leads to cell reversal and reorientation (Hugdahl *et al*., 1988; Xie *et al*., 2011; Bubendorfer *et al*., 2014; Lele *et al*., 2016).

**FIGURE 1:**
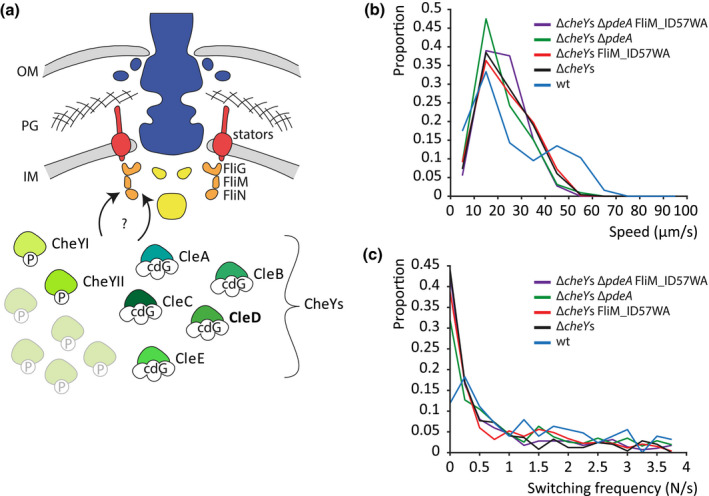
Many CheY homologs modulate *Caulobacter crescentus* swimming behavior. (a) Schematic representation of the flagellar motor structure of *C. crescentus* and its modulation by different homologs of the chemotactic response regulator CheY. CheY‐homologs interact in their phosphorylated (P) or c‐di‐GMP (cdG)‐bound form with the flagellar switch protein FliM of the C ring. (b) Probability distribution of mean run speed (b) and directional switching frequencies (c) of swimming trajectories of different *C. crescentus* strains in a pseudo‐2D environment recorded at 16 frames per second [Colour figure can be viewed at wileyonlinelibrary.com]

A recent study revealed a stable interaction between several CheY‐like proteins and the flagellar motor in the relatively thin, polar flagellated freshwater bacterium *Caulobacter crescentus* (Nesper *et al*., 2017). Its characteristic asymmetric cell division leads to the formation of two different daughter cells: a surface attached “stalked” cell and a flagellated “swarmer” cell. Swarmer cells can disperse from biofilms, allowing the attachment on a new surface, and thus, the colonization of new environments. The small signaling molecule cyclic‐di‐GMP is known to orchestrate the transition from motile to sessile lifestyles in many species (Jenal *et al*., 2017). The *C. crescentus* genome encodes 12 CheY homologs. In pusher mode, when the cell swims straight forward, its flagellar motor rotates by default in a CW direction. Binding of the major CheY protein involved in *C. crescentus* chemotaxis, CheYII (Nesper *et al*., 2017), reverses rotation to CCW, causing the cell to swim backward (Koyasu and Shirakihara, 1984). Other CheY homologs were shown to interact with the N‐terminal binding motif of FliM upon binding to c‐di‐GMP (Figure 1 A). These Cle proteins (CheY‐like c‐di‐GMP effectors) bind to the C ring in addition to phosphorylated CheY and are preparing the flagellar motor for surface attachment. A subset of the Cle proteins was implicated in surface sensing; others are reducing motor speed and promote smooth swimming upon surface encounter (Nesper *et al*., 2017). The protein CleD inhibits motility at high c‐di‐GMP conditions, enabling rapid surface attachment and biofilm formation. The interaction with FliM was strong enough to observe c‐di‐GMP‐dependent colocalization of GFP‐tagged versions CleD. It even allowed co‐purification of FliM‐CleD complexes (Nesper *et al*., 2017). Such a stable interaction between an effector protein and the flagellar motor presents the ideal model system for electron cryotomography (cryoET) and subtomogram averaging (STA).

While the intracellular signaling molecules responsible for switching of rotational direction are well‐studied in diverse bacteria (Toker and Macnab, 1997; Bren and Eisenbach, 1998; Szurmant *et al*., 2003; Ahn *et al*., 2013; Henderson *et al*., 2020), the molecular mechanisms of action of effectors binding to the C ring are unclear. Transient interactions such as CheY binding to the flagellar motor are difficult to study with conventional structural biology approaches. Although the binding interface of CheY and FliM is known, the specific stoichiometry and three‐dimensional arrangement of FliM in the C ring are unresolved (Kim *et al*., 2017). While it is possible to isolate basal bodies and even C rings in *S. enterica* (Thomas *et al*., 2006; Sakai *et al*., 2019), it is difficult to study their transient interactions with relatively small effector proteins. It is even more challenging to study the in vivo interactions between effector proteins and the bacterial flagellar motor (Sourjik and Berg, 2002; Di Paolo *et al*., 2016). CryoET offers a remedy, visualizing protein complexes in situ within flash‐frozen cells. In a process called STA, details can be subsequently resolved by averaging many identical particles such as flagellar motors (Zhao *et al*., 2014; Rossmann and Beeby, 2018). Because cryoET resolutions are inversely proportional to sample thickness, the transient interaction of few CheY molecules is difficult to capture by cryoET. This becomes possible when focusing on targets at the polar regions of thin cells such as *C. crescentus*.

In this study, we examine the molecular architecture of the flagellar motor and specifically the C ring of *C. crescentus* when all *cheY* genes are deleted. This provides additional information about the flagellar motor structure of *C. crescentus*, which is an important model organism for motility and biofilm formation. Subsequently, we overexpressed one CheY‐like protein, CleD, in the CheY‐gutted strain and imaged the putative binding location of this protein in detail. This improves our understanding of the mechanism of action of these c‐di‐GMP‐binding proteins, and we also present the first structure of a CheY‐homolog bound to the flagellar motor at nanometer resolution. This demonstrates the potential of cryoET and STA to not only acquire in situ structures, but also capture interactions of multi protein complexes with effector molecules.

## 2 RESULTS

### 2.1 Deletion of all *cheY* genes leads to straight swimming without reversals

To image binding of a single effector to the C ring, we first sought to remove all FliM‐binding proteins so that we could subsequently reintroduce a single effector to study in isolation. We deleted all 12 *cheY* genes encoded in the genome (Figure 1a) of *C. crescentus* CB15, generating the strain CB15 *ΔCC0440 ΔCC1364 ΔCC2249 ΔCC3100 ΔCC3155 ΔCC0432 ΔCC0437 ΔCC0588 ΔCC0591 ΔCC0596 ΔCC3258 ΔCC3471* (CB15 *ΔcheY*s). Single‐cell tracking experiments revealed that this strain was still able to swim, though at a lower speed than the wildtype (wt) (Figure 1b). In contrast to the wt, Δ*cheY*s mutant cells failed to reverse their swimming direction, confirming that all effector proteins which can induce rotational switching were indeed deleted (Figure 1c). The swimming pattern also did not change when in addition the c‐di‐GMP specific phosphodiesterase PdeA was deleted to increase cellular c‐di‐GMP concentrations. Mutating the putative CheY‐binding motif in FliM (FliM_ID57WA) (Figure S1a) in the Δ*cheY*s background strain also had no effect on the switching rate. These results show that the Δ*cheY*s strain indeed lacks all components involved in motor switching via binding to the N‐terminal CheY‐binding motif. This provides the basis for structural studies of specific CheY‐like effector proteins bound to the flagellar C ring.

### 2.2 Structure of the bare CW‐locked *C. crescentus* C ring in situ

**FIGURE 2:**
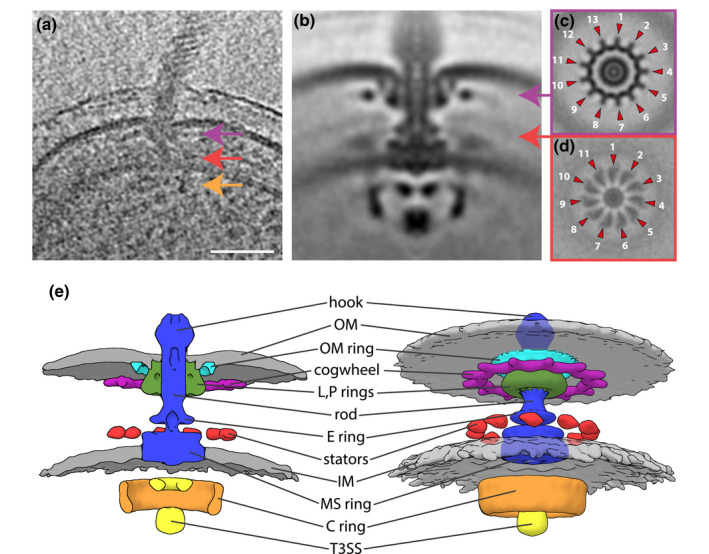
Electron cryotomography and subtomogram averaging of *C. crescentus* Δ*cheY*s mutant provides an improved structure of the alphaproteobacterial flagellar motor. (a) Tomographic slice through a flagellated cell pole of *C. crescentus* CB15 Δ*cheY*s with colored arrows indicating the periplasmic cogwheel (magenta), the stator region (red) and the C ring (orange). Scale bar represents 50 nm. (b) 100 nm × 100 nm slice through C13‐symmetrized subtomogram average of the *C. crescentus* CB15 Δ*cheY*s flagellar motor (c and d) 100 nm × 100 nm horizontal slices in the cogwheel plane of a C13‐symmetrized STA (c) and the stator plane of a C11‐symmetrized STA (d) of the *C. crescentus* CB15 Δ*cheY*s flagellar motor. Magenta (c) and red (d) arrows indicate the approximate location of the slice in (b). (e) Isosurface renderings as cross‐section (left) and side view (right) of the C13‐C11 fused structure [Colour figure can be viewed at wileyonlinelibrary.com]

We next used the Δ*cheY*s strain to understand the molecular architecture of the C ring without bound effector protein. CryoET imaging of the Δ*cheY*s mutant revealed a similar cell morphology as previous tomography studies on *C. crescentus* (Chen *et al*., 2011; Guerrero‐Ferreira *et al*., 2011; Bharat *et al*., 2017) (Figure S2a,b). However, in the *che*Ys deletion strain many of the stalks were also flagellated forming a bulge, which is big enough to accommodate the flagellar motor (Figure S2a). Despite the improved signal‐to‐noise ratio in tomograms of the thin flagellated stalks, subsequent STA was exclusively performed on flagellar motors from the non‐stalked pole, since diffusion barriers between the polar stalk extension and the cell body (Schlimpert *et al*., 2012) might prevent cytoplasmic effector proteins to access motors situated in the stalk. Only few cell poles were flagellated and the flagellar motors were often located at subpolar positions, making acquisition of sufficient particles challenging. To this end, 278 motors from the pole opposite the stalk were extracted from the tomographic data and used to generate a structure of the *ΔcheY*s strain (Figures 2a, S2b).

Although the *C. crescentus* motor had the same conserved overall core structure as previously described (Chen *et al*., 2011), the higher resolution allowed us to discern finer details. The C ring, with a diameter of 35 nm, exhibited the known peanut‐shape in a cross‐section (Figure 2b). The thicker lower part is probably composed of FliN, a thinner middle part might be formed by FliM and a slightly thicker upper section is commonly assigned to FliG. This improved structure of the *C. crescentus* Δ*cheY*s C ring, therefore, allowed us to lay the groundwork for an in‐depth molecular understanding of the C ring without the interference of CheY‐like effectors.

### 2.3 The *C. crescentus* motor features 11 putative stator complexes and a symmetry‐mismatched 13‐fold symmetric periplasmic ring

We also observed additional periplasmic ring structures in the *C. crescentus* motor. A ring above the MS ring with a diameter of 17 nm corresponds to the previously reported E ring (Stallmeyer *et al*., 1989). A 30‐nm wide ring encircles the P and L rings beneath the outer membrane, which we name the outer membrane (OM) ring, and a second ring with a diameter of 40 nm located just below the first ring. Thirteen 7.5‐nm long spoke‐like structures extend from the wider ring, giving it a cog‐like appearance with a total outer diameter of 55 nm in the horizontal central slice (Figures 2c, S3a,b). This structure is described as “cogwheel” in Figure 2e.

Another density just above the inner membrane, corresponding to the location of the stator complexes, was clearly visible in a vertical section of the STA (Figure S3a,b). However, similar to the previous structure (Chen *et al*., 2011), this density appeared fuzzy and the stator symmetry could not be resolved. Since the 13‐fold symmetric cogwheel seemed to have driven the rotational alignment during STA, we speculated that the stator ring might exhibit a different rotational symmetry. The custom mask used for the alignment of the subtomograms in this STA included the whole periplasmic and cytoplasmic section of the motor (Figure S3c). Thus, we attempted to redo the alignment, masking out the stator region (Figure S2g) and exclusively allowing rotational searches around the y‐axis. Concurrent classification of the original dataset provided a structure composed of 112 motors with a ca. 50 nm wide ring in the stator plane exhibiting clear 11‐fold symmetry (Figures 2d, S2e,f). The STAs of the other two classes displayed no apparent stator symmetry. This suggests a maximal stator occupancy of 11 stators per motor, the fewest stator complexes visualized in a flagellar motor to date. Since this density is poorly resolved, we assumed that most motors might only be occupied by fewer stator units. However, the binding locations of the engaged stator units seem to be discrete. This allowed us to build a composite model of the flagellar motor of *C. crescentus* exhibiting two different symmetries, 13 for the cogwheel and 11 in the stator plane (Figure 2e).

### 2.4 CleD overexpression leads to a bulkier C ring, indicating an outside‐facing binding site for CheY‐homologs

We were now poised to ask where CheY‐homologs bind the C ring. Since we suspected that basal expression of these individual proteins and physiological c‐di‐GMP concentrations were insufficient to saturate effector binding to the flagellar motor, we decided to overproduce CleD at high intracellular c‐di‐GMP concentrations (Δ*pdeA*). CleD was selected because of its higher molecular mass and its ability to form stable complexes with FliM (Nesper *et al*., 2017). Most of the CheY residues known to be involved in interaction with FliM in *E. coli* are conserved in CleD (Figure S1b). To this end, we constructed a Δ*cheY*s Δ*pdeA* strain ectopically overexpressing CleD under the control of a xylose‐inducible promotor (pMT375 CleD‐GFP). GFP was fused to the C‐terminus of CleD to confirm its subcellular localization to the flagellated cell pole and to increase its overall mass to generate more density in the STA.

The observation that this strain showed lower motility on semi‐solid agar plates (Figure S4a) and that CleD‐GFP localized to the pole opposite the stalk (Figure S4b) confirmed that this CleD variant is able to interfer with flagellar motility at high c‐di‐GMP concentrations (Nesper *et al*., 2017). Thus, this overexpression strain ensured maximal occupation of CleD binding sites, increasing the chances to successfully detect additional densities derived from bound effector proteins.

We then used this strain to determine the structure of the C ring with bound CleD. 150 motors were averaged as indicated above, generating an independent motor structure without using the Δ*cheY*s structure as reference for alignment (Figure S5a). This strain and all other strains used for generating subsequent STAs frequently formed two flagellar motors at the pole opposite the stalk. Since both motors formed filaments, we assume that they are fully assembled and functional (Figure S2c).

**FIGURE 3:**
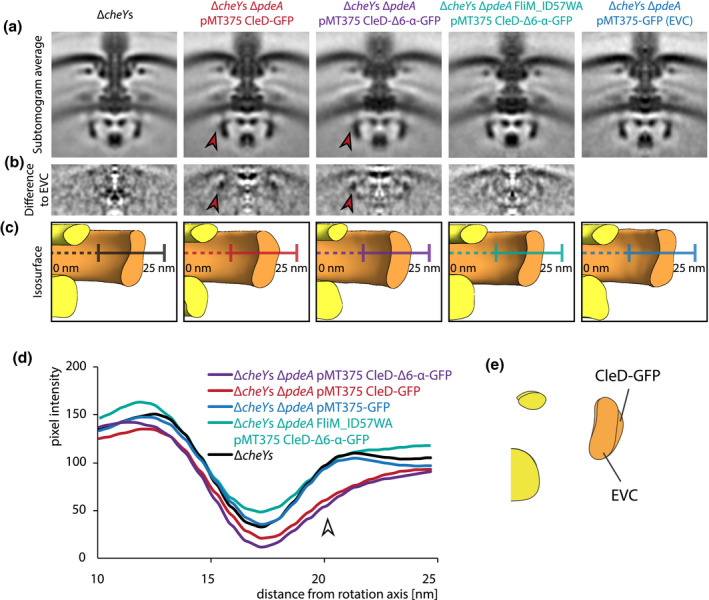
Comparison of flagellar motor structures reveals an additional density at the outer lobe of the flagellar C ring if CleD‐GFP is bound. (a) 100 nm × 100 nm vertical slices of C100‐symmetrized STAs of the flagellar motor from Δ*cheY*s, Δ*cheY*s Δ*pdeA* pMT375 CleD‐GFP, Δ*cheY*s Δ*pdeA* pMT375 CleD‐Δ6‐α‐GFP, Δ*cheY*s Δ*pdeA* FliM_ID57WA pMT375 CleD‐Δ6‐α‐GFP and Δ*cheY*s Δ*pdeA* pMT375‐GFP. Red arrow indicates the presence of an additional density. (b) Vertical slices through 3‐dimensional difference maps obtained by signal subtraction of STAs of Δ*cheY*s, Δ*cheY*s Δ*pdeA* pMT375 CleD‐GFP, Δ*cheY*s Δ*pdeA* pMT375 CleD‐Δ6‐α‐GFP and Δ*cheY*s Δ*pdeA* FliM_ID57WA pMT375 CleD‐Δ6‐α‐GFP with the STA empty vector control Δ*cheY*s Δ*pdeA* pMT375‐GFP. All STAs were 2 nm low pass filtered and aligned prior to signal subtraction. Additional densities are represented by darker intensities in the difference map. (c) Isosurface rendering of the C ring region of Δ*cheY*s, Δ*cheY*s Δ*pdeA* pMT375 CleD‐GFP, Δ*cheY*s Δ*pdeA* pMT375 CleD‐Δ6‐α‐GFP, Δ*cheY*s Δ*pdeA* FliM_ID57WA pMT375 CleD‐Δ6‐α‐GFP and Δ*cheY*s Δ*pdeA* pMT375‐GFP. All STAs were 2 nm low pass filtered and aligned before generation of the isosurface. The same signal threshold was applied for all STAs. Width of all panels is 100 nm. (d) Intensity plot of pixel intensities of vertical STA slices along the cross section of C rings represented by colored lines in (c). The arrow marks the putative binding location of CleD at the outer lobe of the C ring. All STAs were 2 nm low pass filtered and aligned prior to intensity blot generation. (e) Overlay of stylized isosurface cross sections of the C ring region of the Δ*cheY*s Δ*pdeA* pMT375‐GFP and the Δ*cheY*s Δ*pdeA* pMT375 CleD‐GFP strain. STAs were 8 nm low pass filtered and aligned prior to isosurface generation [Colour figure can be viewed at wileyonlinelibrary.com]

Comparison of the motor structure obtained from the Δ*cheY*s Δ*pdeA* pMT375 CleD‐GFP strain and the subtomogram average of the motor from the Δ*cheY*s strain revealed a subtle but distinct thickening at the outer lobe of the middle part of the C ring (Figure 3a). While the width of the bottom part of the C ring density remains around 4 nm in all structures, the characteristic constriction of the C ring was not visible in the CleD overexpression strain. This strain exhibits a significant thickening and an additional density at the outer rim of the C ring. Exact measurements of the thickening of the C ring were difficult to obtain due to an additional diffuse density at the outer lobe of the C ring.

We hypothesized that this fading signal from the major density results from the conformational flexibility of the C‐terminal GFP‐tag, which was fused to CleD by a flexible linker sequence. To increase the rigidity of the C‐terminal part of this fusion protein, we redesigned the linker region of the construct by deleting the last six amino acid residues which are disordered according to PSIPRED (Jones, 1999) (Figure S1b)and fused it to GFP via a rigid α‐helix linker sequence (Chen *et al*., 2013). The expression of this protein on the plasmid pMT375 CleD‐Δ6‐α‐GFP was supposed to boost the signal in this area. Subsequent, reference‐free STA with 197 subtomograms revealed a structure with a similarly diffuse density outside the C ring. However, crucially, and reinforcing our proposed visualization of CleD binding Δ*cheY*s Δ*pdeA* pMT375 CleD‐GFP, the bulky outside facing density remained present (Figures 3a, S5c).

These results strongly supported the hypothesis that the additional density at the outer lobe of the C ring is derived from bound CleD. However, the relatively modest density increase required a negative control experiment to exclude the possibility that this density is caused by experimental artifacts. STA on a Δ*cheY*s Δ*pdeA* strain with a plasmid expressing only GFP was chosen to control for GFP alone contributing to the additional density. This strain carrying the plasmid pMT375‐GFP was treated and induced in the same way as the two CleD‐GFP overexpression strains. 114 motors from this strain were used to generate a reference‐free STA. The central slice of the C ring structure from the empty vector control did not show the extra density resulting in the same peanut‐shape as the Δ*cheY*s C ring with a width of around 3 nm at the constriction (Figures 3a, S5g). This confirms that the extra density is not caused by experimental artifacts from the xylose‐inducible plasmid system, free GFP or other bound effector proteins responding to high c‐di‐GMP concentrations.

We further sought to test whether our additional density corresponded to recent findings that CleD interacts with FliM in the C ring via a conserved, N‐terminal binding sequence which resembles the CheY binding motif in other flagellar systems (Nesper *et al*., 2017). We tested the prediction that our putative CleD additional density would disappear if this CheY‐binding domain were disrupted by mutating Ile_57_‐Asp_58_ in FliM to Trp_57_‐Ala_58_ (Nesper *et al*., 2017) (Figure S1a) in our *C. crescentus* Δ*cheY*s Δ*pdeA* pMT375 CleD‐Δ6‐α‐GFP background. The resulting structure composed of 133 motors resembled the C ring from the Δ*cheY*s and the empty vector control STA (Figures 3a, S5e). An extra density or a thickening of the C ring as seen in the structures with overexpressed CleD and wt FliM was not apparent. This finding supports the model that the N‐terminal binding domain of FliM is associated with the additional CleD density.

**FIGURE 4:**
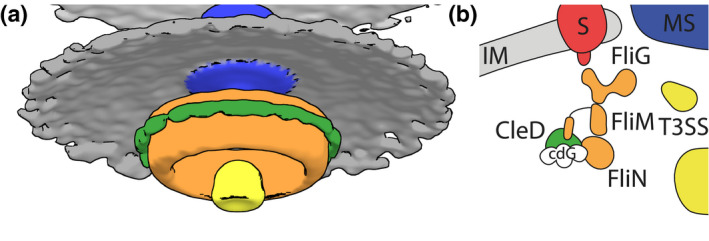
Electron cryotomography enables in situ visualization of conformational changes in a CheY‐bound flagellar motor. (a) Stylized difference map in isosurface representation of the STAs of the Δ*cheY*s Δ*pdeA* pMT375‐GFP and the Δ*cheY*s Δ*pdeA* pMT375 CleD‐GFP strains with a defined threshold of 2.7 σ using the same color code as in B. STAs were 2 nm low pass filtered and aligned prior to isosurface generation. (b) Schematic model illustrating the binding of c‐di‐GMP‐bound CleD at the outer lobe of the flagellar C ring in *C. crescentus* [Colour figure can be viewed at wileyonlinelibrary.com]

Difference maps between the negative control and the two CleD‐GFP overexpression strains allowed us to better visualize the additional density. This additional density was missing in the difference map, if the negative control structure was compared to the Δ*cheY*s or the Δ*cheY*s Δ*pdeA* FliM_ID57WA pMT375 CleD‐Δ6‐α‐GFP structure (Figure 3b). Central slices of isosurfaces of all structures, which were generated by applying the same threshold, indicated the location of the bulge derived from overexpressing CleD variants (Figure 3c). Comparing density plots of the C ring further visualizes the extra density and the fading signal likely caused by the GFP‐tag on the outside of the C ring (Figure 3d). Finally, a difference map of radially averaged CleD‐GFP and empty vector structures in isosurface representation with a defined threshold of 2.7 σ helps to visualize the location of the CleD binding interface (Figure 4a). Together, this data strongly supports a model in which CleD binds to the outside of the C ring likely via the N‐terminal CheY‐binding motif of FliM.

## 3 DISCUSSION

In this study, we aimed to image the structure of the *C. crescentus* C ring when a CheY‐like effector protein is bound. This was achieved by acquiring the structure of the *C. crescentus* flagellar motor when all CheY homologs were deleted using cryoET and STA. We then compared this improved structure with structures of strains where a CheY‐like binding protein was selectively overexpressed. This novel approach to visualizing client protein binding made it possible to detect a CheY homolog binding to the outside of the C ring.

Upon overexpression of GFP‐tagged CleD, we see the main increase in density in the middle and upper part of the C ring (Figures 3c,e, 4a). Previous studies in *E. coli* suggested that CheY binds to the N‐terminus of FliM (Bren and Eisenbach, 1998; Lee *et al*., 2001) but also interacts with FliN (Sarkar *et al*., 2010). It is therefore possible that the arrangement of CleD at the C ring differs from the arrangement of phosphorylated CheY and the C ring. The c‐di‐GMP binding ARR domain from CleD could reach toward the interface between FliM and FliG/MotA, where it might interfere with the torque generating mechanism of the rotor. This would explain why CleD binding to FliM has a different effect on motor function than phosphorylated CheY. Our results also clearly show that the N‐terminal binding site of FliM in *C. crescentus* is likely to face toward the outside of the C ring, which in the future might help to build a more reliable model of the C ring (Figure 4b). It also further suggests that the interaction between CleD and FliM must be relatively strong. Future studies could explore the binding affinity and stoichiometry of the CleD:FliM interaction. This might be important in understanding the binding competition with the other 12 CheY homologs in *C. crescentus*. The introduction of a rigid α‐helix linker sequence between CleD and GFP did not drastically improve the imaged electron densities. It is therefore possible that the GFP tag is still too flexible to contribute significantly to the additional density. Nevertheless, it served as an experimental replicate to further strengthen our identified binding location of CleD. Together these results provide a structural basis to understand the function of molecular motors and how they respond to extracellular stimuli.

The small size of the CheY protein and its transient interaction with the C ring made it previously impossible to detect such proteins in situ. There is a possibility that the additional density is, in part, due to local movements of FliM coupled to the association of CleD. That would, however, require large rearrangements of FliM, which would also probably disrupt FliN binding. The detailed analysis of the STAs using signal subtraction and pixel intensity plots and the unprecedented use of control experiments convincingly show that the additional density indeed corresponds to CleD.

To our knowledge, this is the first in situ structure of a molecular machine with a bound effector protein. This provides a basis for future experiments which might allow to map the interaction site between CheY‐homologs and FliM in greater detail. Increasing the number of motors in *C. crescentus*, as previously performed in gammaproteobacteria (Zhu *et al*., 2017; Zhu *et al*., 2019) or engineering a corresponding FliM‐CleD system in a thinner bacterium with more motors could allow collection of an increased amount of data. This would also answer the question if the observed CleD/FliM arrangement is comparable to other CheY homologs also in other organisms. We collected all of our data on a 200 kV electron microscope without energy filter and a side‐entry cryoholder that produces images with indiscernible Thon rings, preventing CTF correction, and therefore, limiting our resolution. Collection on improved equipment and subsequent CTF‐correction would greatly boost our resolution. This will open up new possibilities for using cryoET and STA of bacterial flagellar motors and clearly demonstrates the potential of these techniques to not only image in situ structures, but also interactions with client proteins.

The Δ*cheY*s strain was used to acquire a structure of a simplified flagellar motor without any bound accessory proteins. The reduced swimming speed of the CheY‐gutted strain compared to the wildtype might resemble the speed reduction of Δ*cleA*, Δ*cleA‐E* or Δ*cheYI* mutants in previous experiments (Nesper *et al*., 2017). However, the specific cause of the reduced swimming speed of the Δ*cheY*s strain is unknown. Directional switching, however, was absent beyond noise due to the lack of FliM‐binding chemotaxis response regulators. These experiments confirm that probably all potential c‐di‐GMP‐dependent FliM‐binding effector proteins modulating motor function were deleted in the Δ*cheY*s mutant. This drastically reduces the complexity of motor‐associated effector network in the Δ*cheY*s mutant, allowing the isolated functional and structural examination of specific effector proteins. Together, this suggests that our Δ*cheY*s motor structure probably presents the molecular arrangement of a C ring without any c‐di‐GMP‐dependent or chemotaxis‐related, bound effector proteins.

Although the resolution of our *C. crescentus* motor structures remain modest, they are a considerable improvement upon previously published structure (Chen *et al*., 2011), revealing three previously unseen periplasmic ring structures as well as bound stator complexes, adding to our picture of the structural diversity in flagellar motors, including those from gammaproteobacteria (Zhu *et al*., 2017; Kaplan *et al*., 2019; Ferreira *et al*., 2019), epsilon‐ and deltaproteobacteria (Beeby *et al*., 2016; Chaban *et al*., 2018) and spirochetes (Qin *et al*., 2018). The composition and the function of these three periplasmic ring structures remain elusive. The presence of a “larger outer disk” had previously been hinted at (Stallmeyer *et al*., 1989), reporting enlarged 31‐ or 34‐nm wide L rings, depending on the purification method, possibly corresponding to the 30 nm OM ring and/or the 40/55 nm cogwheel from our structure. The presence of two distinct rings and the variable stability of these structures under different purification conditions suggest that these rings are composed of at least two proteins. Outer membrane‐associated rings found in other organisms (Beeby *et al*., 2016; Zhu *et al*., 2017; Chaban *et al*., 2018; Zhu *et al*., 2018; Kaplan *et al*., 2019; Ferreira *et al*., 2019) but so far have not been conclusively allocated a function. Previous studies in gammaproteobacteria associated the presence of outer rings with higher motor speed (Kaplan *et al*., 2019), penetration of the outer layer of the bacterial envelope (Zhu *et al*., 2018) or ejection of flagella (Ferreira *et al*., 2019). We could not, however, identify homologs of the putative major components of the H ring from polarly flagellated gammaproteobacteria or the basal disk in epsilonproteobacteria in *C. crescentus*. Because the 13‐spooked cogwheel is located 5 nm away from the outer membrane, we speculate that it is unlikely to directly interact with the outer membrane. What other functions these ring structures might have remains to be understood.

The additional smaller ring above the *C. crescentus* MS ring, not found in gammaproteobacteria*,* may be the enigmatic E ring suggested to be involved in flagellar ejection (Stallmeyer *et al*., 1989; Kanbe *et al*., 2005). Interestingly, all currently known flagellar motor structures from epsilon‐ and deltaproteobacteria possess a similar, additional E ring structure above the MS ring. Phylogenetic analysis of core rod proteins revealed that these species are all in the same clade, indicating that the E ring might be an inherent feature in alpha‐, epsilon‐ and deltaproteobacteria (Figure S5). However, we failed to identify suitable common candidate proteins, which might be responsible for the formation of this ring. Nevertheless, the E ring represents yet another evolutionary elaboration in flagellar motors.

The 11‐fold symmetry of the stator ring reveals new insights into the torque‐generating part of the motor. This small stator ring with the fewest stator units encountered so far reflects the low torque condition this fresh water bacterium experiences in its environmental niche (Li and Tang, 2006; Beeby *et al*., 2016). This is also the first ever described stator symmetry in a motor where no scaffolding structures like MotXY in polar‐flagellated gammaproteobacteria or PflAB in epsilonproteobacteria are present. This suggests discrete binding positions for the stator complexes independent of any scaffolding proteins. These locations might therefore be determined exclusively by interaction with FliG and steric effects. A mean occupancy of eleven stators was also predicted for *E. coli* (Khan *et al*., 1988; Reid *et al*., 2006; Leake *et al*., 2006) but so far stator symmetry has not been observed in situ. This could, in part, be due to decreased numbers of incorporated stator units under low motor loads (Lele *et al*., 2013). *C. crescentus* might exhibit maximal stator occupancy also at lower load. Together, this suggests that the *C. crescentus* stators might be less dynamic than their *E. coli* counterparts and are optimized for low torque conditions.

## 4 EXPERIMENTAL PROCEDURES

### 4.1 Bacterial strains and growth conditions

*C. crescentus* CB15 strains were cultivated in rich medium (peptone yeast extract; PYE) under vigorous shaking at 30°C (Ely, 1991). For pMT375 plasmid‐carrying strains, the medium was supplemented with Tetracycline (5 µg/ml for solid; 2.5 µg/ml for liquid media) and induced with 0.3% of D‐(+) xylose. Motility behavior of *C. crescentus* was carried out on PYE softagar plates (0.3% of agar) supplemented with Tetracycline and Xylose if necessary.

### 4.2 Strain constructions

Marker‐less deletions in *C. crescentus* were generated using the standard two‐step recombination sucrose counter‐selection procedure based on pNPTS138‐derivatives. *E. coli* S17.1 was used to transfer suicide plasmids by conjugation into *C. crescentus* strains (Ely, 1991). Transformation of cells with pMT375 was performed by electroporation. The bacterial strains, plasmids and oligos used in this work are summarized in Table S1, S2 and S3.

### 4.3 Fluorescence microscopy

Bacterial strains for fluorescence microscopy were grown to an OD600 of 0.3 and induced for 2 hr with 0.1% of xylose. 4 µl of cells were spotted on 1% of agarose pads. Phase contrast and fluorescence microscopy images were taken at 30°C with a pco.edge sCMOS camera on a Delta Vision Core microscope (GE Healthcare) equipped with a UPlan FL N 100× oil objective (Olympus). For fluorescence microscopy, the GFP filter set (Ex 461‐489 nm, Em 525‐50 nm) was used with illumination at 100% of intensity and an exposure time of 250 ms.

### 4.4 Swimming speed tracking

Cultures were grown until they reached an OD660 of about 0.15‐0.2. A sample was then diluted to a target density of 0.002 OD660 and 10 μl were added in a µ‐Slide 18 Well (uncoated) (cat.: 81826, ibidi GmbH). The slide was immediately placed under a microscope (Eclipse Ti2; Nikon Instruments Europe B.V.) with a 40× objective. For high‐throughput tracking of swimming bacteria, we used a previously developed method (Taute *et al*., 2015). Briefly, this method allows 3D tracking of 2D microscopy movies: by performing cross‐correlations of observed diffraction patterns with a reference library created using 1 μm silica beads, it is possible to determine the *z*‐position of swimming bacteria. Bright field movies were recorded at 15 fps, for a duration of 30 s per sample. The focal plane during acquisition was inside the well at 80 μm from the slide glass bottom. We used the provided software (Taute *et al*., 2015) to track bacteria, and then, measured speed and switching frequencies with an in‐house developed Matlab code.

### 4.5 CryoET sample preparation

All *C. crescentus* strains were cultivated to mid‐exponential phase (OD 0.3‐0.5) and induced as necessary. Cells were subsequently pelleted and resuspended to an OD600 of ~20. Ultraufoil R2/2 grids (200‐mesh) (Quantifoil Micro Tools GmbH) were glow‐discharged for 60 s at 10 mA and a solution of 10 nm colloidal gold in 1% of BSA was pelleted and mixed with 25 μl cells immediately before plunge freezing. A 2.5 μl droplet of this sample solution was applied to the glow‐discharged electron microscopy grid; the grid was blotted and plunge‐frozen into a liquid ethane‐propane mixture using a Vitrobot plunge‐freezing robot (FEI Company) with a wait time of 60 s, a blot time of 5 s, and blot offsets −3 mm. Grids were stored under liquid nitrogen until data collection.

### 4.6 CryoET of *C. crescentus*

Tilt series were collected on a 200‐kV FEI Tecnai TF20 FEG transmission electron microscope (FEI Company) equipped with a Falcon II direct electron detector camera (FEI Company) using a Gatan 914 cryoholder. Tilt series were recorded from an angle of −54° to +54° with an increment of 3° and a collected defocus between −3 μm and −4 μm using Leginon automated data‐collection software (Suloway *et al*., 2009) at a nominal magnification of 29,000x and were binned four times to final pixel size of 0.7006 nm. Cumulative doses of ~120 e^−^/Å^2^ over the tilt series were used. Overnight data collection was facilitated by the addition of a 3 L cold‐trap Dewar flask and automated refilling of the Dewar cryo‐holder triggered by a custom‐written Leginon node interfaced with a computer‐controlled liquid nitrogen pump (Norhof LN2 Systems).

### 4.7 STA

Tomograms were reconstructed automatically using RAPTOR (Amat *et al*., 2008) and the IMOD package (Kremer *et al*., 1996). Low‐defocus images were low‐pass filtered to remove data beyond 3.5 nm. Positions of flagellar motors in tomograms were initially aligned manually along their rotational axes using different custom masks (Figure S3c,g). The particle estimation for electron tomography (PEET) package (Nicastro, 2006) was used for iterative subtomogram extraction, fine alignment, classification and averaging. Resolution was estimated by Fourier shell correlation (FSC) (Figures S3d,h, S5b,d,f,h).

### 4.8 STA data visualization

For comparison of different structures, STAs were normalized and low‐pass filtered as required using the EMAN (Ludtke *et al*., 1999) and EMAN2 software package (Tang *et al*., 2007). STAs were subsequently aligned in PEET (Nicastro, 2006). Difference maps were generated using the EMAN package (Ludtke *et al*., 1999). Isosurfaces were created using UCSF chimera (Pettersen *et al*., 2004). Intensity plots were made in ImageJ. Radial averaging was performed using C11, C13 or C100 symmetrization.

### 4.9 Phylogenetics

Phylogenetic trees were generated essentially as described previously (Chaban *et al*., 2018). The protein sequences of four flagellar rod proteins FlgB, FlgC, FliE, FliF were aligned, trimmed and concatenated. Bootstrap sampling was done 1,000 times with a termination criteria of 10,000 generations without a topology improvement of 0.01 in the lnL score. Bootstrap support was converted to percentage and added to the base trees.

## CONFLICT OF INTEREST

The authors declare no conflict of interest.

## AUTHOR CONTRIBUTIONS

FMR, IH, UJ and MB conceived and designed the study; FMR, IH and MS collected the data; FMR, IH, MS, UJ and MB analyzed the data; FMR, IH, UJ, MS and MB wrote the manuscript; and all authors approved the manuscript.

## Supporting information

Supplementary Material

## Data Availability

The subtomogram averaging data is available on EMDB (Δ*cheY*s C13, EMD‐10943; Δ*cheY*s C11, EMD‐10945; Δ*cheY*s C100, EMD‐10949; Δ*cheY*s Δ*pdeA* pMT375 CleD‐GFP, EMD‐10950; Δ*cheY*s Δ*pde* pMT375 CleD‐Δ6‐α‐GFP, EMD‐10955; Δ*cheY*s Δ*pdeA* FliM_ID57WA pMT375 CleD‐Δ6‐α‐GFP, EMD‐10956; Δ*cheY*s Δ*pdeA* pMT375‐GFP, EMD‐10957). All other data that support the findings of this study are available from the corresponding author upon reasonable request.
