# Supplementary material for: In situ structure of the Caulobacter crescentus flagellar motor and visualization of binding of a CheY‐homolog
Source: Mol Microbiol. 2020 May 25;114(3):443–53. doi: 10.1111/mmi.14525 (PMC7534056; doi:10.1111/mmi.14525)
Supplement: Supplementary file 1 — Supplementary Material [file MMI-114-443-s001.pdf]

Supplementary information

Supplementary Figures and Tables

***In situ* structure of the *Caulobacter crescentus* flagellar motor and visualization of binding of a CheY-homolog**

Florian M. Rossmann [1], Isabelle Hug [2], Matteo Sangermani [2], Urs Jenal [2] and Morgan Beeby[1]

[1]Department of Life Sciences, Imperial College London, London, United Kingdom

[2]Focal Area of Infection Biology, Biozentrum of the University of Basel, Basel, Switzerland

Address correspondence to: Morgan Beeby, 502 Sir Ernst Chain Building, South Kensington Campus, London SW7 2AZ, United Kingdom; Tel: +44 (0)20 7594 5251; Email: m.beeby@imperical.ac.uk

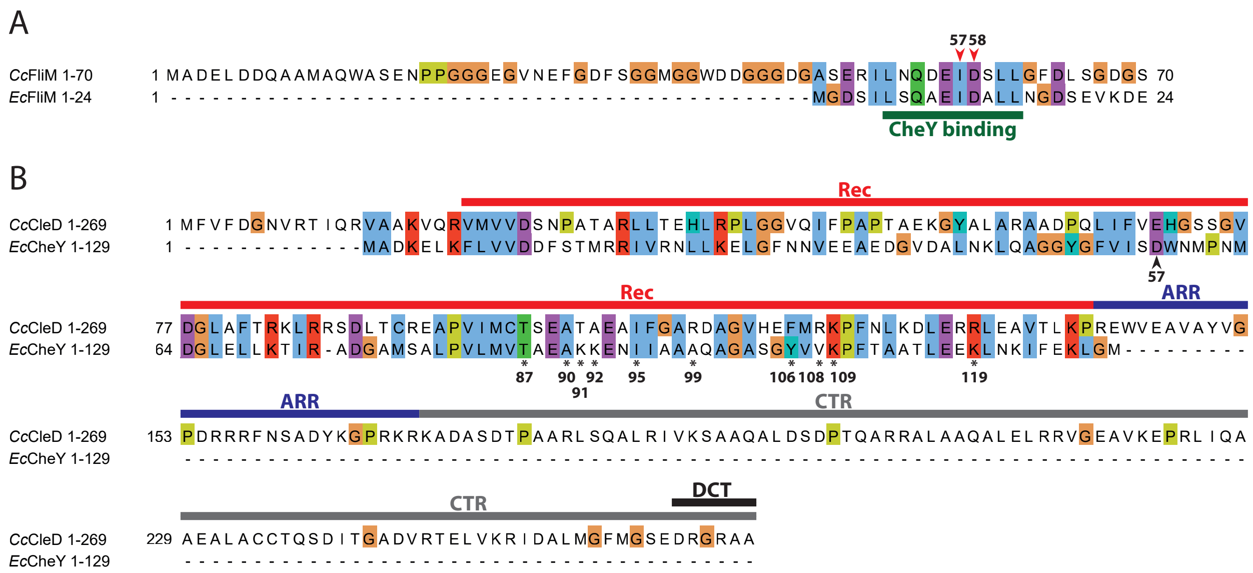

Fig S1. Sequence alignments of CheY/CleD and FliM from *Escherichia coli* and *Caulobacter crescentus*. (A) Sequence alignment of the N-terminus of FliM from *C. crescentus* and *E. coli*. *Cc*, *C. crescentus*; *Ec*, *E. coli*. Residues mutated in the FliM_ID57WA strain are marked with a red arrow. Numbers above the marked residues indicate their position in the *C. crescentus* CleD protein sequence. (B) Alignment of protein sequences of CleD from *C. crescentus* and CheY from *E. coli*. The segments of the receiver domain (Rec), Arg-rich region (ARR), C-terminal region (CTR) and six disordered C-terminal residues (DCT) are highlighted. The *E. coli* CheY phosphorylation site is marked with an black arrow. Residues involved in interaction of CheY with FliM in *E. coli* are marked with asterisks (*). Numbers below the marked residues indicate their position in the *E. coli* CheY protein sequence. Adapted from Nesper *et al.* 2017. The color code indicates conservation and identity of amino acids in the alignment.

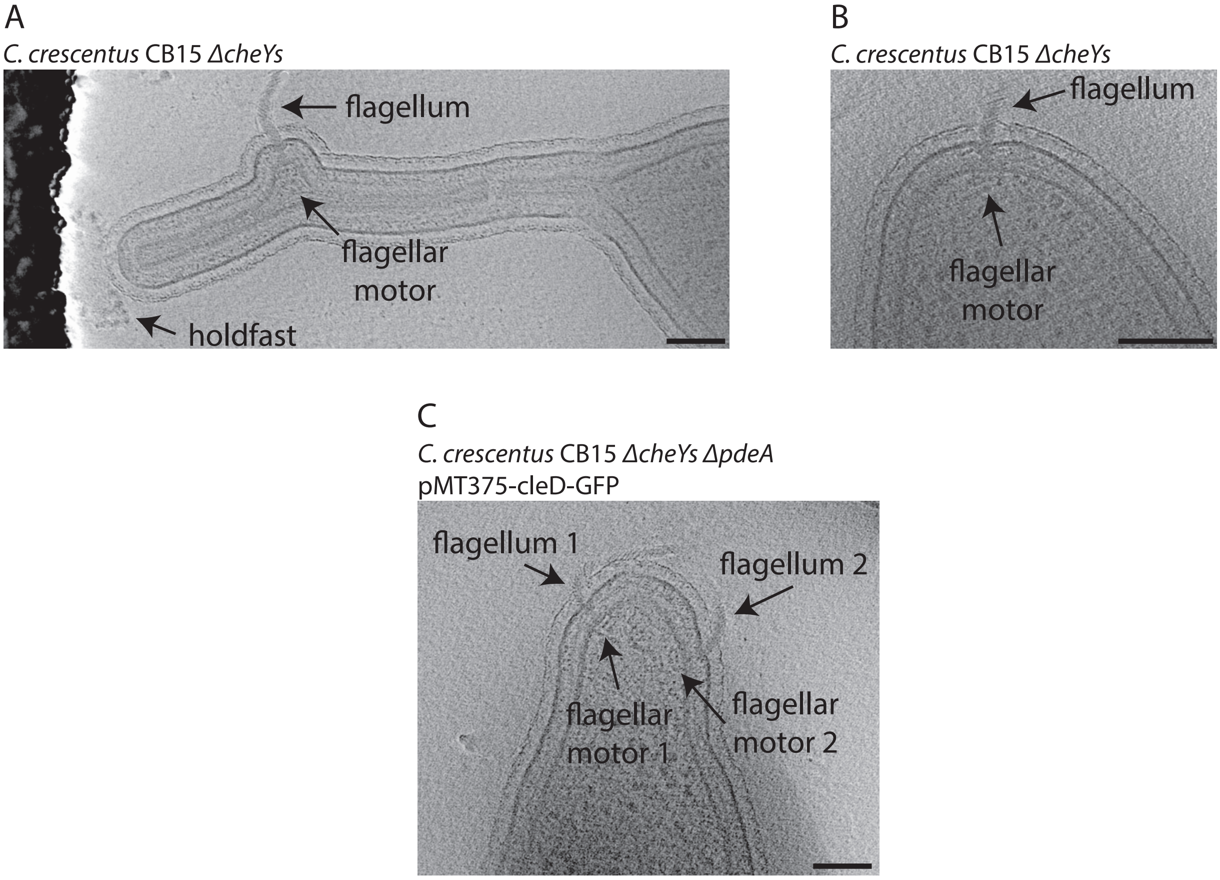

Fig S2. Representative, tomographic slices of flagellated *C.crescentus* cell poles. Tomography slices of (A) flagellated stalks and (B) flagellated cell poles in a Δ*cheY*s mutant. (C) Cell pole of a Δ*cheY*s Δ*pdeA* pMT375 GFP strain harbouring two intact flagella. The scale bars represent 200 nm.

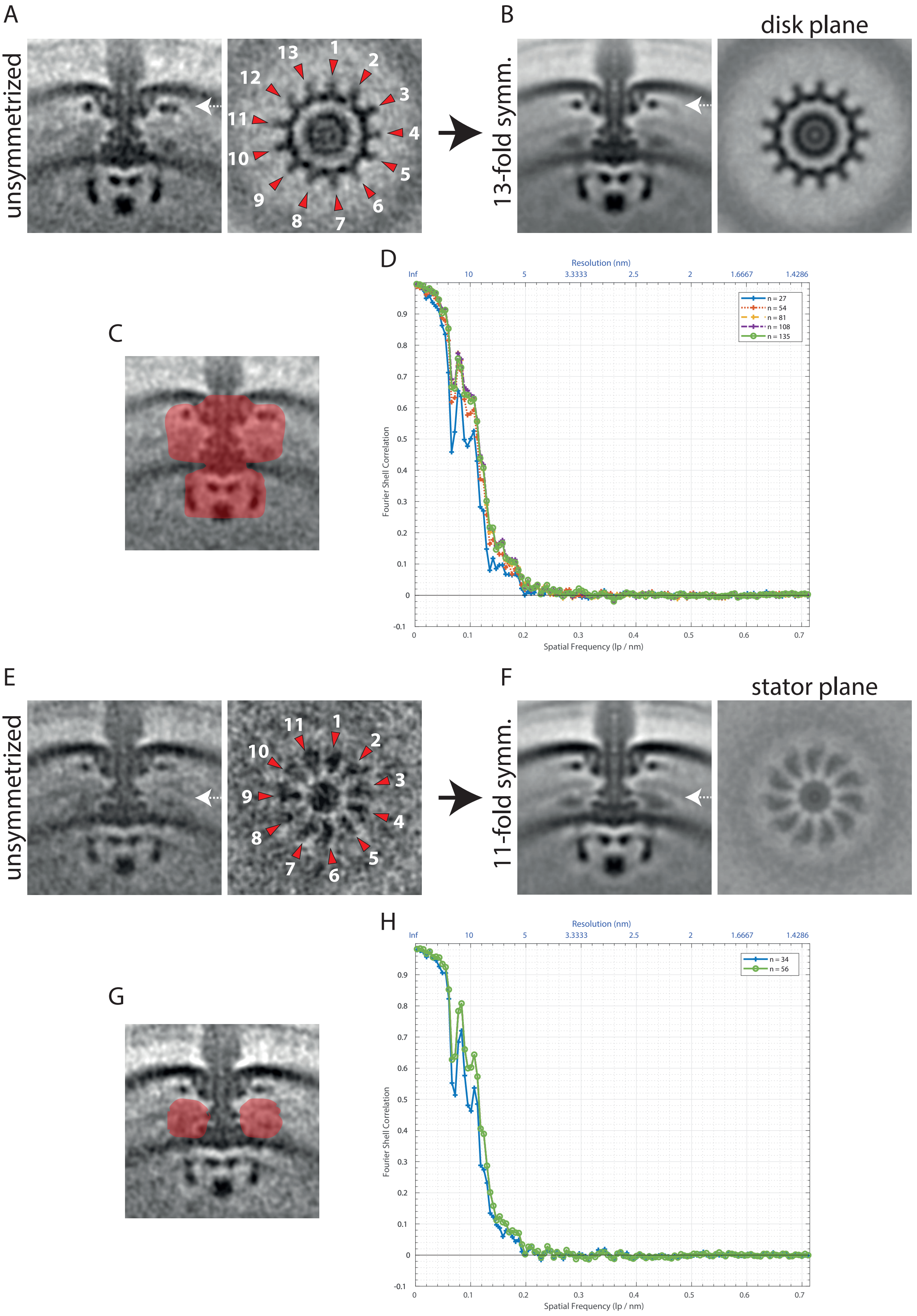

Fig S3. Slices and FSC curves of subtomogram averages of the *C. crescentus* Δ*cheY*s flagellar motor using two different masks. (A) Unsymmetrised and (B) C13-symmetrised vertical, central slice (left panels) and horizontal slice through the cogwheel plane (right panels) from a Δ*cheY*s STA using a custom mask (C) incorporating the entire motor higlighted in red. (D) FSC curve of the STA shown in A. (E) Unsymmetrised and (F) C11-symmetrised vertical, central slice (left panels) and horizontal slice through the stator plane (right panels) of a Δ*cheY*s STA after classification using a donut-shaped, custom mask (G) incorporating only the stator region higlighted in red. (H) FSC curve of the STA shown in E. All STAs are represented as 100 nm x 100 nm slices.

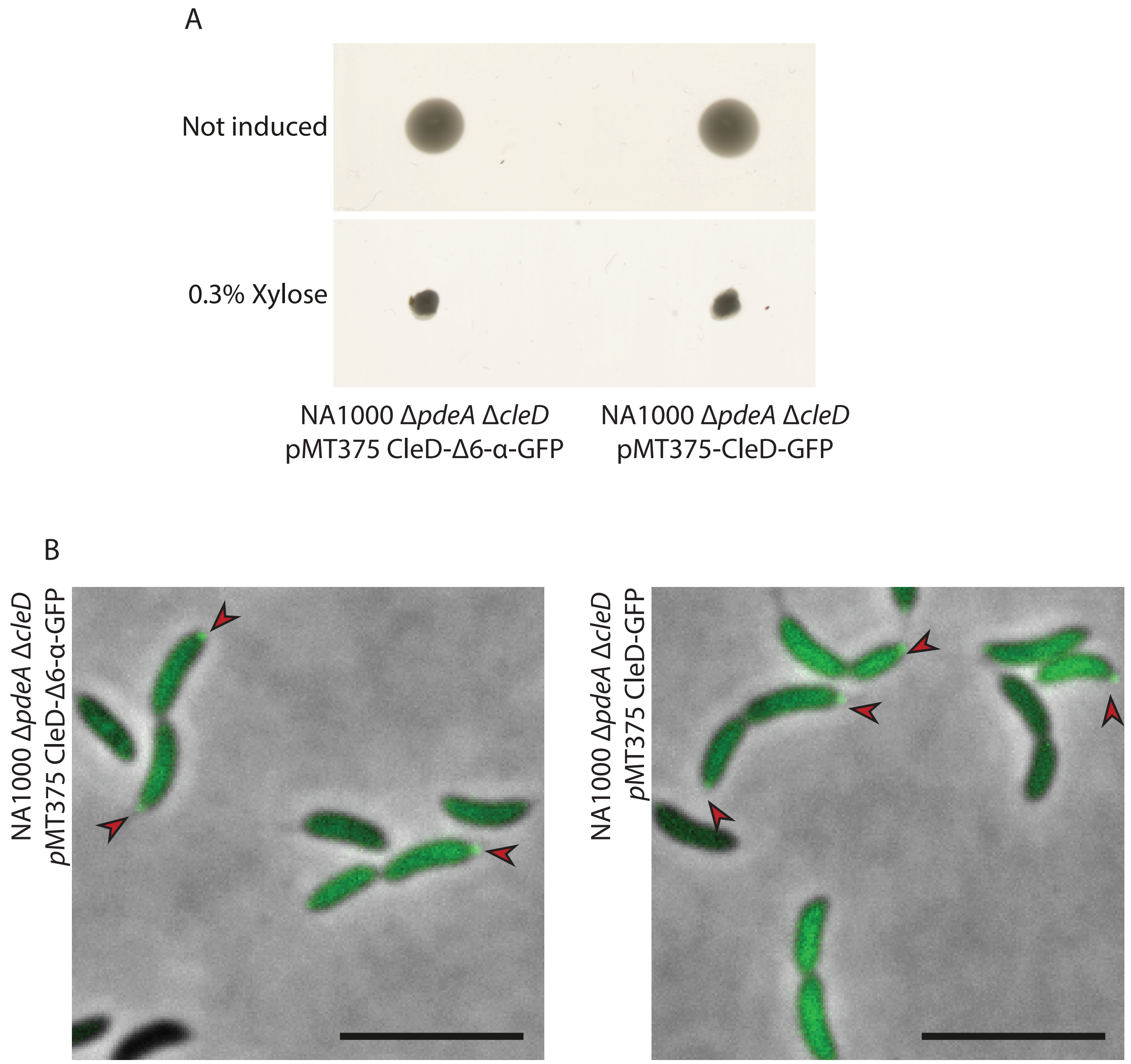

Fig S4. Functional control of pMT375 CleD-Δ6-α-GFP construct. (A) NA1000 Δ*pdeA* Δ*cleD* pMT375 CleD-Δ6-α-GFP and the published (Nesper et al., 2017) control strain NA1000 Δ*pdeA* Δ*cleD* pMT375 CleD-GFP were spotted on a soft agar plate with and without 0.3% xylose. The strain harbouring pMT375 CleD-Δ6-α-GFP suppressed later expansion under high c-di-GMP condition as efficient as the previously tested pMT375 pMT375 CleD-GFP construct. (B) Polar localisation of GFP-tagged CleD was present both in NA1000 Δ*pdeA* Δ*cleD* pMT375 CleD-Δ6-α-GFP and the control strain NA1000 Δ*pdeA* Δ*cleD* pMT375 CleD-GFP, suggesting the overproduced CleD-Δ6-α-GFP is fully functional. The scale bar represents 5 µm.

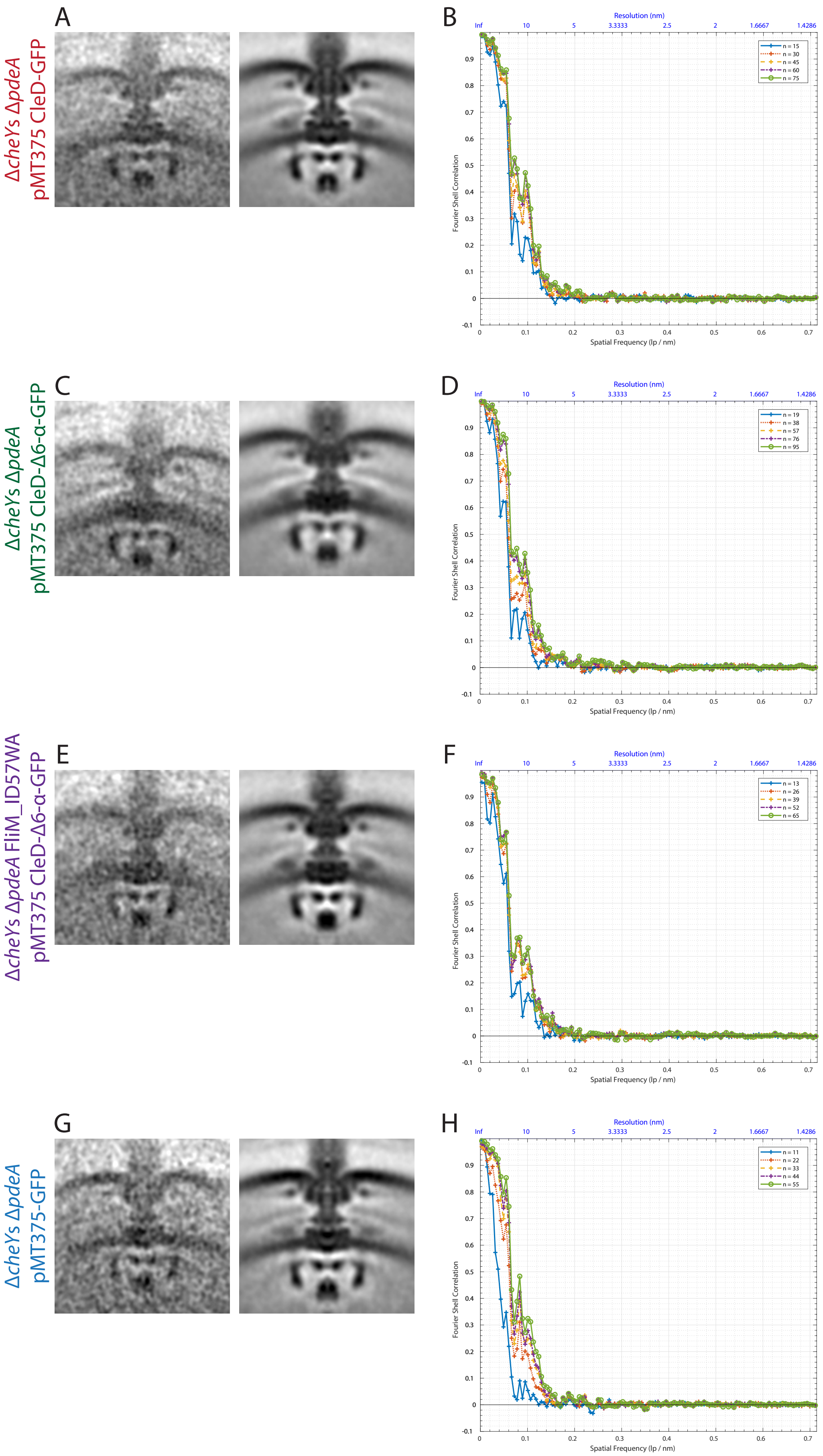

Fig S5. Subtomogram averages and FSC curves of STAs of different *C. crescentus* flagellar motors. (A) Unsymmetrised (left panel), C100-symmetrised (right panel) vertical, central slice and (B) FSC curve of the *C. crescentus* Δ*cheY*s Δ*pdeA* pMT375 CleD-GFP flagellar motor. (C) Unsymmetrised (left panel), C100-symmetrised (right panel) vertical, central slice and (D) FSC curve of the *C. crescentus* Δ*cheY*s Δ*pdeA* pMT375 CleD-Δ6-α-GFP flagellar motor. (E) Unsymmetrised (left panel), C100-symmetrised (right panel) vertical, central slice and (F) FSC curve of the *C. crescentus* Δ*cheY*s Δ*pdeA* FliM_ID57WA pMT375 CleD-Δ6-α-GFP flagellar motor. (G) Unsymmetrised (left panel), C100-symmetrised (right panel) vertical, central slice and (H) FSC curve of the *C. crescentus* Δ*cheY*s Δ*pdeA* pMT375 GFP flagellar motor. All Subtomogram averages are represented as 100 nm x 100 nm slices.

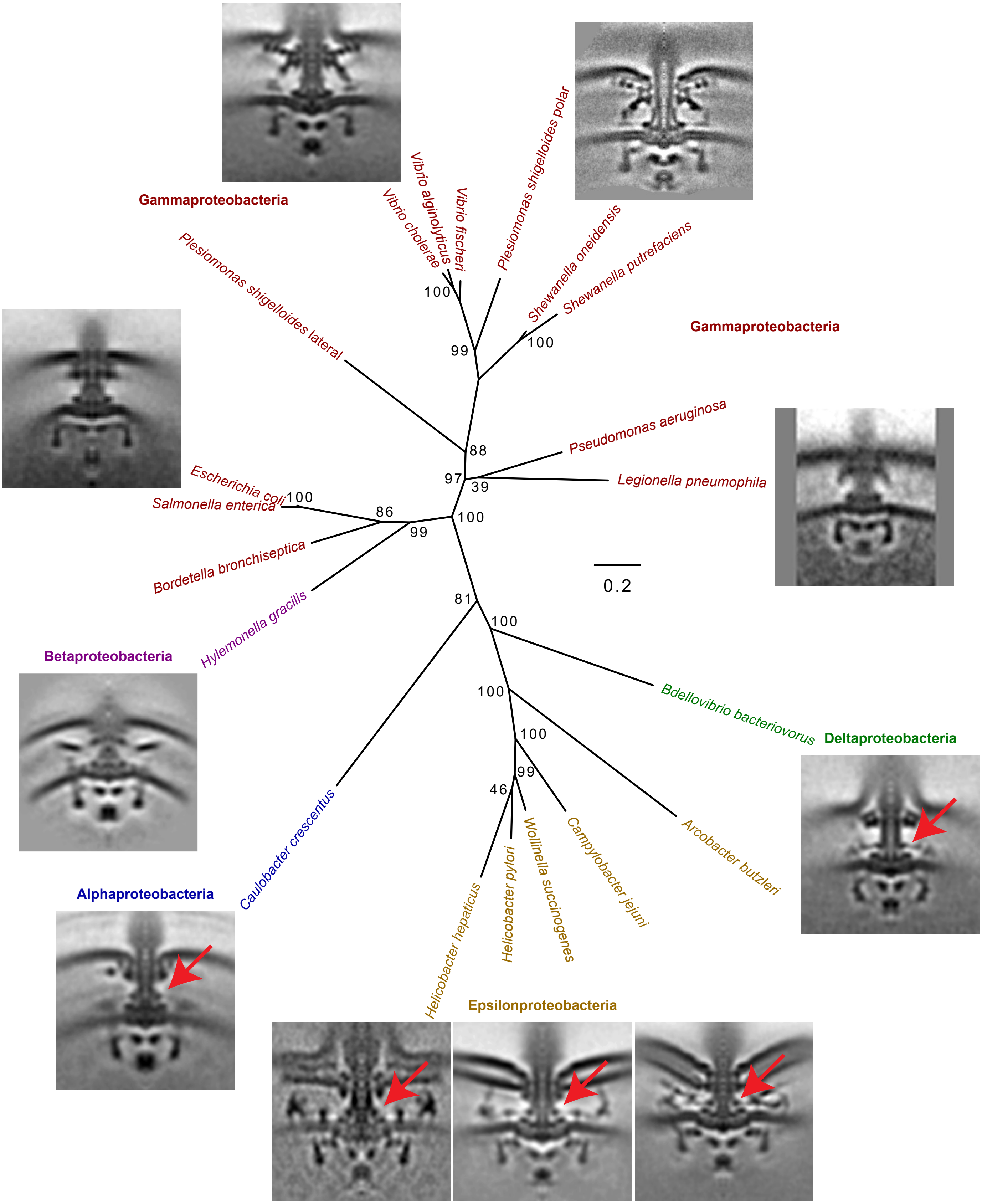

Fig S6. Comparison of the *C. crescentus* Δ*cheY*s flagellar motor structure with other published proteobacterial motor structures. Bootstrapped, unrooted phylogenetic tree of four conserved rod proteins with focus on proteobacteria with published flagellar motor structures. Phylogeny was determined using a concatenation of the core flagellar rod proteins FlgB, FlgC, FliE, FliF. The percent bootstrap support for 1000 replicates is shown at each branching point of the tree. Representative species from alphaproteobacteria are depicted in blue, betaproteobacteria are depicted in purple, gammaproteobacteria are depicted in red, deltaproteobacteria are depicted in green and epsilonproteobacteria are depicted in yellow. Central, vertical slices of STAs from following organisms (in clockwise order starting in the top right corner): *Plesiomonas shigelloides* (Ferreira *et al.*, 2019); *Legionella pneumophila* (Kaplan *et al.*, 2019); *Bdellovibrio bacteriovorus* (Chaban *et al.*, 2018); *Campylobacter jejuni* (Beeby *et al.*, 2016); *Wolinella succinogenes* (Chaban *et al.*, 2018); *Helicobacter pylori* (Qin *et al.*, 2016); *Caulobacter crescentus* (this study); *Hylemonella gracilis* (Chen *et al.*, 2011); *Salmonella enterica* (Beeby *et al.*, 2016); *Vibrio fischeri* (Beeby *et al.*, 2016). Red arrows point at the putative E ring. All Subtomogram averages are represented as 100 nm x 100 nm slice.

Table S1: Bacterial strains that were used in this study

Strain	Name	Genotype and description	Source
	Escherichia coli		
1	DH10B	F-*mcrA* D(*mrr-hsd* RMS- *mcrBC*) f80d*lacZ*M15D*lacX*74 *endA*1 rec1deoR D(*ara*, *leu*)7697 *araD*139 *galU nupG rpsL thi prohsd*+ *recA* RP4-2-Tc::Mu-Tn7	(Evinger and Agabian, 1977)
2	S17.1	F-, lambda (-), *thi*, *pro*, *recA*, restriction (-) modification (+),RP4 derivative integrated into the chromosome with Tet::Mu, Km::T7	(Evinger and Agabian, 1977)
	Caulobacter crescentus		
3	CB15	C. crescentus wild type ATCC 19089 *Caulobacter vibrioides* LOT:3967454	(Stove Poindexter and Cohen-Bazire, 1964)
4	NA1000	WT; Synchronizable laboratory strain of CB15 (CB15N)	(Evinger and Agabian, 1977; Muir and Gober, 2001)
5	NA1000 Δ*pdeA* Δ*cleD*	UJ5832, Markerless in frame deletion of *cleD* (CC3100) and *pdeA* (CC3396) in NA1000	(Nesper *et al.*, 2017)
6	CB15 Δcc0440 ΔCC1364 ΔCC2249 ΔCC3100 ΔCC3155	UJ8851, Markerless in frame deletion of *cleA* (CC0440), c*leB* (CC1364), *cleC* (CC2249), *cleD* (CC3100) and *cleE* (CC3155) in CB15	(Nesper *et al.*, 2017)
7	CB15 ΔCC0440 ΔCC1364 ΔCC2249 ΔCC3100 ΔCC3155 ΔCC0432	Markerless in-frame deletion of *cheYI* (CC0432) in strain 6	This study
8	CB15 ΔCC0440 ΔCC1364 ΔCC2249 ΔCC3100 ΔCC3155ΔCC0432 ΔCC0437	Markerless in-frame deletion of *cheYII* (CC0437) in strain 7	This study
9	CB15 ΔCC0440 ΔCC1364 ΔCC2249 ΔCC3100 ΔCC3155 ΔCC0432 ΔCC0437 ΔCC0588	Markerless in-frame deletion of *cheYIb* (CC0588) in strain 8	This study
10	CB15 ΔCC0440 ΔCC1364 ΔCC2249 ΔCC3100 ΔCC3155 ΔCC0432 ΔCC0437 ΔCC0588 ΔCC0591	Markerless in-frame deletion of *cheYIIb* (CC0591) in strain 9	This study
11	CB15 ΔCC0440 ΔCC1364 ΔCC2249 ΔCC3100 ΔCC3155 ΔCC0432 ΔCC0437 ΔCC0588 ΔCC0591 ΔCC0596	Markerless in-frame deletion of *cheYIVb* (CC0596) in strain 10	This study
12	CB15 ΔCC0440 ΔCC1364 ΔCC2249 ΔCC3100 ΔCC3155 ΔCC0432 ΔCC0437 ΔCC0588 ΔCC0591 ΔCC0596 ΔCC3258	Markerless in-frame deletion of *cheYIII* (CC3258) in strain 11	This study
13	CB15 Δ*cheY*s	Markerless in-frame deletion of *cheYIVc* (CC3471) in strain 12	This study
14	CB15 Δ*cheY*s Δ*pdeA*	Markerless in-frame deletion of *pdeA* (CC3396) in strain 13	This study
15	CB15 Δ*cheY*s FliM_ID57WA	Markerless in-frame substitution of residue Ile 57 and Asp 58 to Trp 57 and Ala 58 in FliM (CC2061) in strain 13	This study
16	CB15 Δ*cheY*s Δ*pdeA* FliM_ID57WA	Markerless in-frame deletion of *pdeA* (CC3396) in strain 15	This study

Table S2: Plasmids that were used in this study

Plasmid	Relevant genotype or phenotype	Source
pNPTS138	*mob*RP4+ *sacB*, suicide plasmid for in frame deletions in *Caulobacter crescentus*, kan^R	D. Alley
pNPTS138-D*cheYI*	Suicide vector for in-frame deletion of *cheYI* (CC0432)	(Nesper *et al.*, 2017)
pNPTS138-D*cheYII*	Suicide vector for in-frame deletion of *cheYII* (CC0437)	(Nesper *et al.*, 2017)
pNPTS138-DCC0588	Suicide vector for in-frame deletion of the *cheY*-homolog CC0588	This study
pNPTS138-DCC0591	Suicide vector for in-frame deletion of the *cheY*-homolog CC0591	This study
pNPTS138-DCC0596	Suicide vector for in-frame deletion of the *cheY*-homolog CC0596	This study
pNPTS138-DCC3258	Suicide vector for in-frame deletion of the *cheY*-homolog CC3258	This study
pNPTS138-DCC3471	Suicide vector for in-frame deletion of the *cheY*-homolog CC3471	This study
pNPTS138-D*pdeA*	Suicide vector for in-frame deletion of *pdeA* (CC3396)	(Abel *et al.*, 2011)
pNPTS138_FliM-ID-WA	Suicide vector for in-frame substitution of residue Ile 57 and Asp 58 to Trp 57 and Ala 58 in FliM (CC2061)	This study
pMT375	xylose-inducible, low copy number overexpression plasmid, pMCS-5, tet^R	(Thanbichler *et al.*, 2007)
pMT375-GFP	Plasmid for xylose-inducible expression of *mGFPmut3*	(Nesper *et al.*, 2017)
pMT375 CleD-GFP	Plasmid for xylose-inducible expression of C-terminally *mGFPmut3*-tagged CleD	(Nesper *et al.*, 2017)
pMT375 CleD-Δ6-α-GFP	Plasmid for xylose-inducible expression of C-terminally *mGFPmut3*-tagged CleD-variant where the last 6 AS (residues 243-248, DRGRAA) were deleted and rigid α-helix linker (Ala Glu Ala Ala Ala Lys Ala) between CleD and *mGFPmut3*	This study

Table S3: Oligonucleotides that were used in this study

Identifier of oligonucleotides	Sequence (5'-3')	Description
11593	TAGTCAGAATTCGCGTTGCTGAAATCGGCG	Primers for generating plasmid pNPTS138-Δcc0588 using overlap PCR with NA1000 genomic DNA as template and EcoRI/HindIII restriction sites for restriction/ligation into pNPTS138.
11594	GATCATGAAAAACAGAGGACTGATATTTGGTAGGCTGCGGCGCCGCGCG	
11595	CGCGCGGCGCCGCAGCCTACCAAATATCAGTCCTCTGTTTTTCATGATC	
11596	ACTAACAAGCTTGACGCCAAGGCTTCCGC	
11597	TAGTCAGAATTCCCAGAAGAACCAGCGCCATC	Primers for generating plasmid pNPTS138-Δcc0591 using overlap PCR with NA1000 genomic DNA as template and EcoRI/HindIII restriction sites for restriction/ligation into pNPTS138.
11598	CGGACGGAGCCAAGAGACAAAGTGACCGCGATGACGGCGC	
11599	GCGCCGTCATCGCGGTCACTTTGTCTCTTGGCTCCGTCCG	
11600	ACTAACAAGCTTCAGGTCGGTCTTGGTTTGCTG	
11601	TAGTCAGAATTCGGAGAGCGGATCGCAGCC	Primers for generating plasmid pNPTS138-Δcc0596 using EcoRI/EcoRV-digested and CIP treated pNPTS138, SOE-PCR with NA1000 genomic DNA as template, and subsequenrt PKN treatment.
11602	CATTATGGAGCCGCAGCATGAGCGCCGCCGTCG	
11603	CGACGGCGGCGCTCATGCTGCGGCTCCATAATG	
11604	ACTAACAAGCTTGATGGGCTCGCCGTTCTG	
11605	TAGTCAGAATTCCCCTGGGCGTCGCTG	Primers for generating plasmid pNPTS138-Δcc3258 using overlap PCR with NA1000 genomic DNA as template and EcoRI/HindIII restriction sites for restriction/ligation into pNPTS138.
11606	GTTCACATTCCGCAGAGGTCGACATGACCACCAGCCGCCTG	
11607	CAGGCGGCTGGTGGTCATGTCGACCTCTGCGGAATGTGAAC	
11608	ACTAACAAGCTTGCGCCCTGACGATCTGGAG	
11609	TAGTCAGAATTCCGACTTCATCAGTCCGGCCAG	Primers for generating plasmid pNPTS138-Δcc3471 using overlap PCR with NA1000 genomic DNA as template and EcoRI/HindIII restriction sites for restriction/ligation into pNPTS138.
11610	GATTGGAGCGCGCCGTGGTGACGCCCGAGGACATGG	
11611	CCATGTCCTCGGGCGTCACCACGGCGCGCTCCAATC	
11612	ACTAACAAGCTTGCAACGCGGCCAGCG	
3615	GCGGCAGCCATATGTTCGTGTTTGACGGCAACGT	Primers for generating plasmid pMT375-CleDΔ6-α-helix-mGFPmut3 (pIH353) using overlap PCR with pMT375-cleD-mGFPmut3 (UJ7383) plasmid DNA as template and NdeI/NcoI restriction sites for restriction/ligation into pMT375-cleD-mGFPmut3.
13179	ACTCGCCTTGGCTGCCGCCTCTGCCTCAGACCCCATGAACCCCATC	
13176	GCAGAGGCGGCAGCCAAGGCGAGTAAAGGTGAAGAACTGTTCACCG	
5332	GGCTGCAGCTAGCTTATTTGTAGAGTTCATCCA	
6543	GTTCCAAGCTTGATTCAGCCTGCGACCAATT	Primers for generating plasmid pNPTS138_FliM-ID-WA using overlap PCR with NA1000 genomic DNA as template and HindIII/BamHI restriction sites for restriction/ligation into pNPTS138.
6544	GCGGATCCGGATGCGCGGAATGTTCTTC	
6545	CATTCTGAACCAGGACGAGTGGGCCAGCCTGCTGGGCTTCGAT	
6546	ATCGAAGCCCAGCAGGCTGGCCCACTCGTCCTGGTTCAGAATG	

References

Abel, S., Chien, P., Wassmann, P., Schirmer, T., Kaever, V., Laub, M.T., *et al.* (2011) Regulatory Cohesion of Cell Cycle and Cell Differentiation through Interlinked Phosphorylation and Second Messenger Networks. *Mol Cell* **43**: 550–560.

Beeby, M., Ribardo, D.A., Brennan, C.A., Ruby, E.G., and Jensen, G.J. (2016) Diverse high-torque bacterial flagellar motors assemble wider stator rings using a conserved protein scaffold. *Proc Natl Acad Sci* **113**: 1–10.

Chaban, B., Coleman, I., and Beeby, M. (2018) Evolution of higher torque in Campylobacter-type bacterial flagellar motors. *Sci Rep* **8**: 97.

Chen, S., Beeby, M., Murphy, G.E., Leadbetter, J.R., Hendrixson, D.R., Briegel, A., *et al.* (2011) Structural diversity of bacterial flagellar motors. *EMBO J* **30**: 2972–2981.

Evinger, M., and Agabian, N. (1977) Envelope associated nucleoid from Caulobacter crescentus stalked and swarmer cells. *J Bacteriol* **132**: 294–301.

Ferreira, J.L., Gao, F.Z., Rossmann, F.M., Nans, A., Brenzinger, S., Hosseini, R., *et al.* (2019) γ-proteobacteria eject their polar flagella under nutrient depletion, retaining flagellar motor relic structures. *PLOS Biol* **17**: e3000165.

Kaplan, M., Ghosal, D., Subramanian, P., Oikonomou, C.M., Kjaer, A., Pirbadian, S., *et al.* (2019) The presence and absence of periplasmic rings in bacterial flagellar motors correlates with stator type. *Elife* **8**: e43487.

Muir, R.E., and Gober, J.W. (2001) Regulation of late flagellar gene transcription and cell division by flagellum assembly in Caulobacter crescentus. *Mol Microbiol* **41**: 117–130.

Nesper, J., Hug, I., Kato, S., Hee, C.-S., Habazettl, J.M., Manfredi, P., *et al.* (2017) Cyclic di-GMP differentially tunes a bacterial flagellar motor through a novel class of CheY-like regulators. *Elife* **6**: e28842.

Qin, Z., Lin, W.-T., Zhu, S., Franco, A.T., and Liu, J. (2016) Imaging the motility and chemotaxis machineries in Helicobacter pylori by cryo-electron tomography. *J Bacteriol* **199**: e00695-16.

Stove Poindexter, J.L., and Cohen-Bazire, G. (1964) THE FINE STRUCTURE OF STALKED BACTERIA BELONGING TO THE FAMILY CAULOBACTERACEAE. *J Cell Biol* **23**: 587–607.

Thanbichler, M., Iniesta, A.A., and Shapiro, L. (2007) A comprehensive set of plasmids for vanillate - And xylose-inducible gene expression in Caulobacter crescentus. *Nucleic Acids Res* **35**: e137.
